# Outcomes of a pilot study in chiropractic practices in Western Australia

**DOI:** 10.1186/s12998-016-0116-9

**Published:** 2016-10-10

**Authors:** Lyndon G Amorin-Woods, Gregory F. Parkin-Smith, Lee Nedkoff, Colleen Fisher

**Affiliations:** 1 Senior Clinical Supervisor, School of Health Professions (Discipline of Chiropractic) Murdoch University, Perth, WA Australia; 2 Murdoch University Chiropractic Clinic, South Street Campus, 90 South Street, Murdoch, WA 6150 Australia; 3 General Practice and Emergency Medicine Registrar, Busselton, WA 6160 Australia; 4 Research Fellow, School of Population Health, Faculty of Medicine, Dentistry and Health Sciences, The University of Western Australia, 35 Stirling Highway (M431), Crawley, Perth, WA 6009 Australia; 5 Head of School, School of Population Health, Faculty of Medicine, Dentistry and Health Sciences, The University of Western Australia, 35 Stirling Highway (M431), Crawley, Perth, WA Australia

## Abstract

**Background:**

This paper reports the quantitative outcomes of a mixed-methods pilot study of the characteristics and demographics of chiropractic practices and patients in Western Australia.

**Methods:**

This was a mixed-methods data transformation model (qualitative to quantitative) pilot study. A non-random sample of chiropractic practices across Western Australia was recruited and data collected anonymously from consecutive new patients using an online platform. Data covered practice and patient demographics and characteristics, alongside quality of life measures. A descriptive quantitative analysis characterised the sample, and the patient population was stratified by main reason for presentation to compare characteristics according to the presence of secondary complaints. Odds ratios were calculated to estimate the odds of a secondary complaint for various combinations of main complaints, from univariate logistic regression models.

**Results:**

Of the 539 registered practitioners in WA in July 2014, 33 agreed to participate, from 20 different practices. Ten participating practices provided data on 325 adult new patients. The recruited practices (metropolitan *n* = 8, regional *n* = 2) had a positive response rate of 79.7 % (*n* = 301 metropolitan and *n* = 24 regional patients), mean age 36.3 years (range 18–74) (53.2 % female). Spinal problems were reported as the main reason for consultation by 67 % and as secondary reasons by 77.2 % of patients. People presented primarily for health maintenance or a general health check in 11.4 %, and as a secondary reason 14.8 %. There were 30 % of people below societal norms for the SF-12 Physical Component Score (mean 47.19, 95 % CI; 46.27–48.19) and 86 % for the Mental Component Score (mean 36.64, 95 % CI; 35.93-37.65), Pain Impact Questionnaire mean scores were 54.60 (95 % CI; 53.32–55.88).

**Conclusions:**

Patients presented to chiropractors in Western Australia with a fairly wide range of conditions, but primarily spinal and musculoskeletal-related problems. A significant proportion of patients had associated, or found to be at risk of, depression. Consequently, there are responsibilities and opportunities for chiropractors with respect to providing care services that include health promotion and well-being education related to musculoskeletal/spinal and mental health. This pilot study supports the feasibility of a future confirmatory study where the potential role of chiropractors in spinal/musculoskeletal health management may be explored.

**Trial registration:**

ACTRN12616000434493: Australian New Zealand Clinical Trials Registry (ANZCTR), Registered 5 April 2016, First participant enrolled 01 July 2014 Retrospectively Registered.

## Background

Around 16 % of Australians (over 3 million people), consult a chiropractor at least once a year [1] at a collective out-of-pocket cost estimated between $905-988 million [2, 3]. According to existing data the majority of these consultations are for spinal and related musculoskeletal (MSK) conditions [4, 5]. Chiropractors are the second-most utilised practitioners (19.1 %) after medical practitioners (22.4 %) for back pain in Australia [5, 6]. The Australian Bureau of Statistics (ABS) and other sources [3] indicate that physiotherapists and chiropractors combined provide approximately 12–15 million consultations in Australia annually [7], and in the allied health sector, these 2 professions alone account for around $2.2 billion in annual Australian health care expenditure [3, 8, 9]. Pro-rata estimates from these data place chiropractors as providing between 1.5–2.0 million patient encounters annually in Western Australia (WA) representing private sector health care expenditure of approximately $75 million. Despite this noteworthy utilisation of chiropractic services, little formal data is available about the characteristics of chiropractic practice, practitioners or patients, especially at a local or regional level. Existing Australian data is either limited or dated, and until recently, there have been no on-going, structured evaluations of chiropractic practice, as found in other healthcare professions [10]. The recently established ACORN project is projected to collect and evaluate data from Australian chiropractic practices, but this project is still evolving [11].

Existing data on chiropractic practice or patients are primarily from Europe, the USA and Canada, with limited studies of satisfactory quality providing insight into chiropractic practice in Australia. In summary the amalgamated findings from these studies [4, 12–24] indicate that the vast majority of patients consult chiropractors for spinal pain, mainly lower back, and to a lesser extent neck and related neuro-musculoskeletal disorders. Patients are predominantly referred to a chiropractor by word-of-mouth (family, friends and acquaintances) and they pay for their care ‘out-of-pocket’. Private health insurers cover chiropractic care to some extent however reimbursements are limited and there are often gap fees payable by the patient. Chiropractic patients in most studies are described as aged 35–45 years, employed mainly in professional, managerial or skilled roles with a slightly higher proportion being female. In most countries there are very low rates of referrals from medical practitioners, with somewhat higher numbers in some Scandinavian countries. The overall impression is that the chiropractic profession has evolved predominantly outside of mainstream healthcare, having developed its own under and post-graduate education, professional research and practice, and is, for the most part, not integrated or reliant on mainstream healthcare via referrals or collaboration. Existing studies are cross-sectional descriptive studies which ipso facto analyse populations rather than individuals. None of the existing studies report using mixed methods, which is noteworthy, since the use of quantitative and qualitative approaches in combination is known to provide a better understanding of these types of research problems than either approach alone [25].

Given previous works that document chiropractic practice are outdated, limited by study design, or geographically unconnected to WA, the initial approach to addressing the gaps in knowledge is to start with an external pilot study and gather local (WA) data using a cost-efficient online platform for data capture [26].

### Aims and objectives

One aim of this pilot study was to offer insights into the characteristics of chiropractic practice and patients in WA which are presented in this paper. Another aim was to provide a critique of the methodology which is presented in a separate paper.

The objectives addressed in this paper were thus:

- Recruit and describe a selection of chiropractic practices and patients across various geographical regions and collect data using an online survey platform;
- Analyse and synthesize the results so to develop insights into the characteristics of chiropractic patients and practices in WA.

## Methods

### Practice recruitment

This study was a prospective, mixed-methods (explanatory) [27], external [28] pilot study with qualitative data transformed to quantitative data during analysis [29]. A non-random convenience sample of chiropractic practices across Western Australia was recruited directly via mail, e-mail or by telephone, based on lists of chiropractors compiled from the public domain and from the register of the Chiropractors Association of Australia (WA Branch). The inclusion criteria for practice recruitment were: a) the chiropractor (s) working within the practice was registered with the Australian Health Practitioners Regulation Agency (AHPRA)/Chiropractic Board of Australia (CBA), and b) the practice was able to provide internet access to allow patient participants to complete the survey (s) privately and anonymously. Practices that expressed an interest to participate (via the practice manager or practice principal) were then provided with information about the study in writing. Initially, 18 practice principals agreed to participate, representing 20 practices and 33 practitioners, from across various geographical locations.

### Orientation procedure

Orientation sessions for the recruited practices were conducted to explain the study protocols. Practitioners and office support staff were invited to attend an orientation session conducted by the investigator in the individual practices. Where this was not feasible, the protocols were explained by telephone and online. A cooling off period of 2 weeks following this session was in place.

### Patient recruitment

Consecutive adult patients (> 18 years age) who self-presented to the participating practices for the first time were provided with a study information sheet and invited to participate. Patients who agreed to participate in the study provided consent (electronically online) when directed to the study questionnaires by the clinic support staff, prior to being seen by the consulting chiropractor. There was no patient screening for entry into the study and the participating chiropractors were blind to the patient’s participation status.

### Consent

All participants-patients, practitioners and support staff-provided consent for involvement in the study either by electronic (patients) or written means (practitioners and support staff).

### Data collection

Quantitative and qualitative data were collected on practices, practitioners and support staff using the online survey platform. Data collected from practice staff were location, age, gender, role, years in the role and feedback on the project (quantitative and qualitative). Quantitative data for analysis on patients were collected using an electronic device (iPad) accessing an online questionnaire situated on a popular survey platform, which patients completed at the clinic. This questionnaire has been published previously [30]. Data collected were age, gender, primary language, occupation, payment source, source of referral (if any), presenting complaint (main and secondary), prior treatment, pre-existing health conditions, medication and supplement use, attendance at other health practitioners, lifestyle choices (smoking and alcohol consumption), and previous use of chiropractic. Human quality of life measures were collected using the SF-12 and Pain Impact-R Questionnaires (Health Outcomes license number: QM023627) [31]. We employed self-reporting by patients which has been shown to be a reliable method of data collection in this context [32, 33]. Each participating practice received a unique secure link to allow access to the survey and the patient was provided with this link by support staff to enable them to log in to the online portal and complete the survey. While the patient was provided with a link, this was not connected to their participation data except to give them entry to the portal. Most domains were configured to require an answer in order to proceed. Support staff kept a paper-based recording of participant number, gender, age, accepting or declining and reasons for declining if applicable. The researcher, who was blind to the data collection, collated the data for analysis.

### Data analysis

#### Qualitative data analysis

Conventional and summative content analyses of qualitative data as described by Hseih and Shannon were undertaken for this study [34]. For the conventional content analysis, responses initially were read individually and coded according to the respondent being practitioner or support staff. Through an open coding process, responses from each group were then read individually and coded according to whether they were a ‘positive’, ‘neutral’ ‘negative’ response or a ‘suggestion’. Each of these categories was then examined individually to ensure internal homogeneity and external heterogeneity and confirm associations between them. The qualitative responses were then quantified and tabulated through a summative content analysis process where responses in each of the categories developed for the conventional content analysis were counted and then expressed as a proportion.

#### Quantitative data analysis

Descriptive quantitative analysis of the variables collected was undertaken using SPSS v21 software to characterise the sample practices, chiropractors and patients. Continuous variables are presented as means, and categorical variables as counts and percentages (proportions). The patient population was stratified by main reason for presentation to compare characteristics according to the presence of secondary complaints. Odds ratios were calculated to estimate the odds of a secondary complaint for various combinations of main complaints, from univariate logistic regression models.

#### Ethical and privacy considerations

Approval to conduct this research was provided by the Human Research Ethics Committee of The University of Western Australia (#RA/4/1/6713). Consecutive numbers were used in each practice as a unique identifier for each individual patient. Codes were assigned to categorise practices, however it was not be possible to identify an individual patient from the code. Additionally, because no identifying information such as patient name, date of birth or address was collected there was no potential for re-identification of data. Data collected from study participants were analysed as aggregated data.

## Results

1. Results describing the participating practices, practitioners and support staff
  There were 539 practitioners registered as of June 2014 with AHPRA in the state of WA, and measures were taken to invite all to be part of the study using membership lists and a database collated by the investigator from the public domain. Of these, 20 practice principals expressed an interest and were provided with information about the study. Eighteen practice principals agreed to participate, representing 20 practices and 33 practitioners. There were eight practices (remote *n* = 1, rural *n* = 3, regional *n* = 1, metropolitan *n* = 3) which either withdrew (*n* = 3) or collected no data (*n* = 5) leaving 10 practices and 26 practitioners actively participating. Reasons for withdrawal included a perceived interruption to patient flow, unforeseen staff and administrative factors or no reason for formal withdrawal or non-collection of data. In addition, some practices collected for less than the full 12 weeks of the study. Compared with all registered chiropractors in Australia, those practitioners who participated were of similar age; mean 35.75 SD ± 10.49 (range 24–55), however with an under-representation of females in this study (20 %) compared with national data (36 %), [35] while all support staff in the study were female with a mean age of 36.4 SD ± 14.68 years (range 18–58) and a mean duration of 3.9 years in their role.
  Practices ranged from a large inner-city practice with multiple practitioners (*n* = 6) to small one person practices operating without any office support staff (Table 1). There was also a wide range for duration in practice for practitioners, ranging from new graduates (first year in practice) to very experienced practitioners of over 20 years. Inner city support staff typically were younger (mean age 33.2 years) and in the role for a shorter time (3.58 years) than their regional counterparts (57.0 years and 8.0 years respectively).

**Table 1:** Characteristics of PIStAChiO practices, practitioners and support staff

Practice location	Practitioners (*n*)	Mean age, years	Gender (Female)	Mean years in role	Support staff (*n*)	Mean age, years	Mean years in role
Inner metropolitan	18		30 %		22		
Mean (SD)	3.6 (1.82)	36.00 (10.36)		10.30 (6.04)	4.4 (3.29)	33.20 (13.69)	3.20 (3.77)
Outer metropolitan	5		50 %	15.25	8		
Mean (SD)	1.67 (0.58)	38.25 (13.18)		15.25 (12.53)	2.67 (2.08)	49.00 (12.54)	3.00 (0.61)
Regional	3				3		
Mean (SD)	1.5 (0.71)	29.50 (7.78)	0 %	3.50 (3.54)	1.5 (2.12)	57.00 (6.78)	8.00 (0.78)
Overall	26		20 %		33		
Mean (SD)	2.6 (1.65)	35.75 (10.49)		10.69 (8.16)	3.3 (2.79)	36.50 (14.68)	3.58 (3.57)

Means per practice

**Fig. 1:**
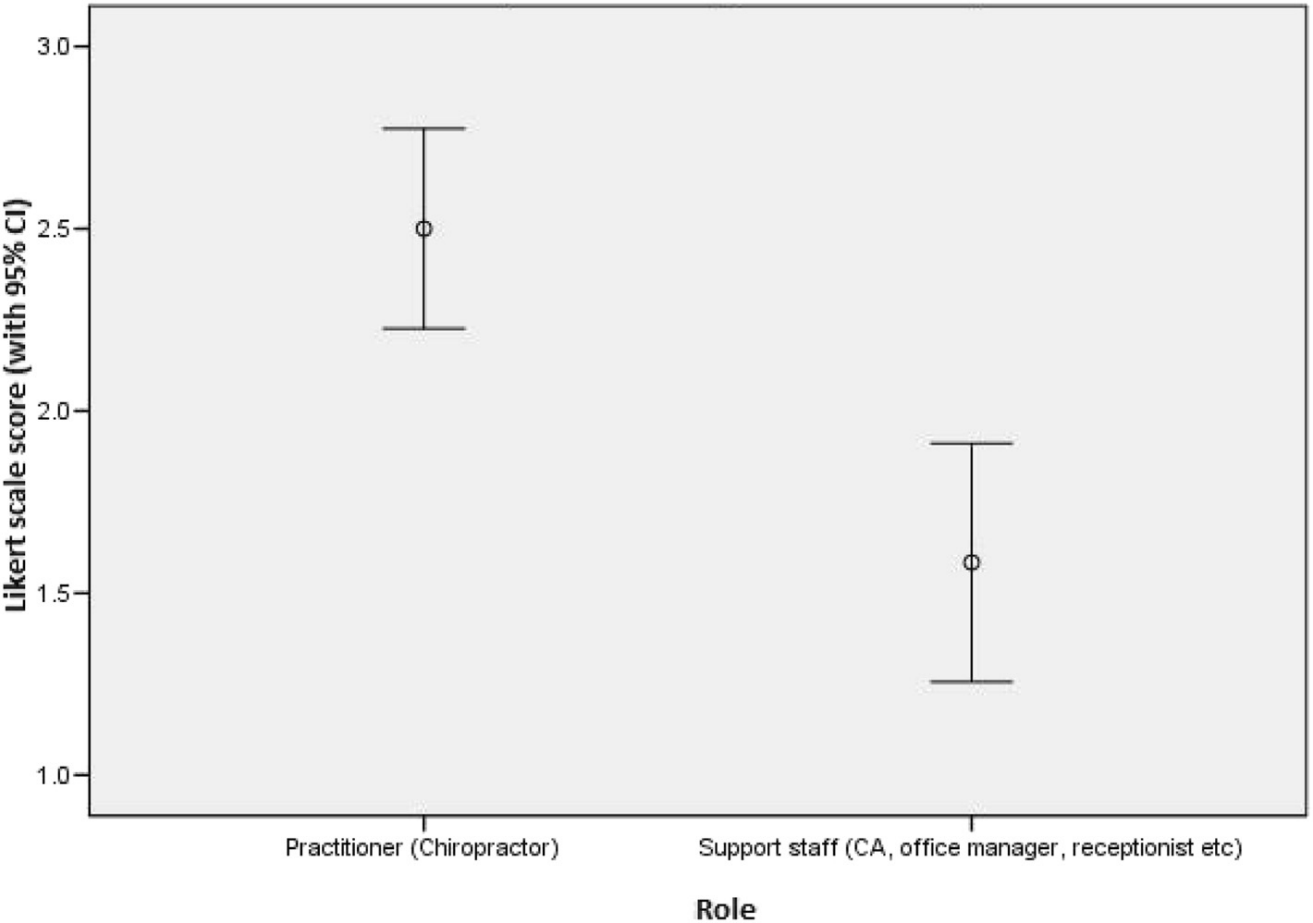
Attitude toward participation in future studies among practitioners and support staff. Mean and 95 % CI for practitioners and support staff (1=Less likely 2=No change 3= More likely)
  Around half of the patient participants (*n* = 164, 50.2 %) were recruited from 1 inner metropolitan multi-practitioner practice. The proportion of patients declining was highest in regional and lowest in inner metropolitan practices and increased in all regions the longer the study continued. In order of frequency, the reasons given for declining were; a lack of time and disinterest (55 % and 54 %), an aversion to technology (24 %), language barrier (14 %), technology (iPad) malfunction (12 %), and visual challenge (4 %) (Fig. 2).

NB: Support staff 100 % female
  Practitioners were significantly (*p* < 0.01) more likely to indicate a positive attitude toward participation in future studies, and to reflect positively about the study than support staff (Fig. 1). Overall, 82 % of all practitioners and support staff were unchanged or more likely to indicate positive feelings toward participation in future research studies.

**Fig. 2:**
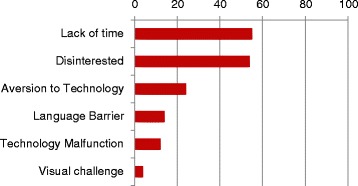
Reasons for patients declining to participate in PIStAChiO study. Proportion of patients declining (%) by reason as recorded by office supports staff Respondents may have indicated more than one reason thus total may >100 %
  Ratings of “good” or better were given to the survey components of 1) background, 2) resources, 3) patient compliance, and 4) access to the team. The components rated the lowest was the impact on clinic operations, rated to be low by both practitioners and support staff. The response rates to the practice feedback survey were: practitioners (*n* = 16; 64 %) and support staff (*n* = 12; 36 %). There were no significant differences in the responses rating the various aspects of the study with the exception of background information.
  The attitudes of 82.1 % of practitioners and support staff were unchanged or more likely to participate in a future study. Qualitative Responses (Fully reported in Appendix 1) from practitioners and practice support staff to the open ended questions were collated and analysed as described by Hsieh and Shannon [34]. Table 2 presents the quantified results from the summative content analysis [34]. The majority of positive responses (90 %) were from practitioners and negative responses (69.33 %) from support staff however the majority (85.71 %) of suggestions for improvements also came from support staff.

**Table 2:** Quantification of qualitative responses by category among practitioners and support staff

	*n* =	% of category	% Practitioners	% Support staff
Positive				
Positive attitude toward research	5	45.45		
Commendation to team	3	27.27		
Low impact on clinic	3	27.27		
Total Positive	11		90	10
Neutral				
No impact due to role	3	75.00		
Technology challenges	1	25.00		
Total Neutral	4		75	25
Negative				
Time	11	32.35		
Inconvenience	5	14.71		
Length of survey	7	20.59		
Technology	1	2.94		
Extra work	5	14.71		
Number of forms	5	14.71		
Total negative	34		30.77	69.33
Suggestions				
Timing	2	25.00		
Challenge	1	12.50		
Disclaimer/length and content of survey	4	50.00		
Wider scope	1	12.50		
Total Suggestions	8		14.29	85.71
Total	57
2. Results describing Chiropractic patients in WA
  Participating practices provided information on 325 new patients. The collecting practices (metropolitan *n* = 8, regional *n* = 2) had an overall response rate of 81.7 % with subsequent sample sizes of 301 patients from metropolitan practices and 24 patients from regional practices.
  Table 3 a and b shows the characteristics of patients who sought chiropractic care. The mean age of patients participating in the study was 36.3 years (SD ± 12.5). Nearly one third were employed as managers or professionals (31.7 %). In 93.2 % of cases, patients paid at least part of the consultation fee. The proportion of smokers were slightly lower than societal norms at 11.7 % [36], and alcohol was reportedly consumed at a level of < 15drinks/week [37] by 96.4 %. Most patients used no prescription (65.5 %) or over the counter (OTC) medication (75.8 %), while just over half (53 %) reported taking no nutritional supplements. The majority (82.7 %) of patients reported no co-morbid conditions. The most common report of a previous diagnosis was that of depression or mental illness (10.2 %). Just under half (48.6 %) had previously sought chiropractic care at other clinics.

**Table 3:** Characteristics of patients (*n* = 325)

(a) Age, demographics, payment and lifestyle	Chiropractic patients
Age, years (mean, SD)	36.3 (12.46)
Gender (female)	Proportion (%) (*n*) 52.3 (170)
Language (Culturally and Linguistically Diverse)^a^	6.5 (21)
Aboriginal Torres Strait Islander	
Yes	0.3 (1)
Prefer not to answer	0.9 (3)
Missing	0.3 (1)
Occupation	
Managers / Professionals	31.7 (103)
Clerical and administrative	12.3 (40)
Technicians and trades	10.5 (34)
Home duties	9.2 (30)
Sales workers	5.8 (19)
Student	7.4 (24)
Community & personal service	4.9 (16)
Machinery operators and drivers	4.6 (15)
Labourers	4.3 (14)
Other	3.7 (12)
Retired	4.0 (13)
Unemployed	1.5 (5)
Source of payment	
Private health insurance	58.8 (191)
Patient paid 100 %	17.8 (58)
Co-payment	16.6 (54)
No Charge	7.7 (25)
Workers compensation	2.2 (7)
Insurance Commission WA (MVA)	0.3 (1)
Department of Veterans’ Affairs	0.3 (1)
Lifestyle	
Smoking (yes)	11.7 (38)
(no)	74.2 (241)
(past)	14.2 (46)
Alcohol consumption, drinks/week	
0	40.9 (133)
1-7	44.6 (145)
7-14	10.8 (35)
> 14	3.7 (12)
Medication/Supplement use	
Prescription/week	
0	65.2 (212)
1	24.0 (78)
2	6.8 (22)
≥ 3	3.9 (13)
Over the Counter	
0	75.7 (246)
1	18.2 (59)
2	4.9 (16)
≥ 3	1.2 (4)
Supplements	
0	52.9 (172)
1	21.2 (69)
2	12.0 (39)
≥ 3	13.8 (45)
(b) Co-Morbidities and Previous Chiropractic	
Co-morbidities	
No other diagnosis	82.7 (273)
Depression	10.2 (33)
Cancer	2.7 (9)
Respiratory Disease	2.7 (9)
Diabetes	2.1 (7)
Cardiovascular Disease	1.8 (6)
Neurological Disorder	0.6 (2)
Any co-morbidity	20.3^b^ (66)
Attendance at previous chiropractor	48.6 (158)

^a^ *Culturally and Linguistically Diverse* = *Non*-*English Speaking Background*

*Categorical variables are shown as percentages and* “*n*” ^b^ *Conditions could be flagged multiple times by each patient*, *therefore total may* ≠ *100* %
  Table 4 shows the referral source of patients and other practitioners consulted for the main complaint. The majority (66 %) of patients came to a chiropractor via word of mouth or recommendation by family or friends. The internet was a fairly common source of patients (22.4 %), yet only 2.1 % listed social media as a referral source. The most common practitioners previously consulted for the main complaint were physiotherapists and medical practitioners.

**Table 4:** Referral source of patients and other practitioners consulted

Source of new patients consulting chiropractors	*n*	Proportion (%)
Word of mouth	111	33.6
Friends or family	107	32.4
Internet	74	22.4
Signage	22	6.8
Referral from other health practitioner	13	3.9
Referral from other source (e.g. lawyer)	9	2.7
Other source	9	2.8
Social Media	7	2.1
Print advertisement	6	1.8
Referral from medical practitioner	6	1.8
Other practitioners previously consulted (for the main complaint)		
No other Health Care Practitioner	174	53.5
Physiotherapist	87	26.4
Medical Practitioner	71	21.5
Massage Therapist	42	12.7
Acupuncturist	12	3.7
Osteopath	9	2.7
Occupational Therapist	5	1.5
Other	4	1.2
  The distribution of problems for which the patients consulted the chiropractors is shown in Table 5. Spinal (any region) problems were the most commonly reported main problems, with 67 % of patients reporting any region of back pain as their main presenting complaint-the majority of patients also reported spinal problems on other regions as a secondary complaint (77.2 %). There was a significant association between reporting of any back pain as the main reason for consultation and also reporting secondary back complaint(s) in one or more different regions (OR 1.84, 95 % CI 1.15, 2.93).

**Table 5:** Main and secondary reasons for presentation to chiropractic clinics

Main reason for presentation	*n* (%)	Secondary reason for presentation†							
		Low Back Problem	Mid Back Problem	Neck Problem	Headache	Shoulder Problem	Hip symptom or complaint	Depression	No other reason
Low back problem	108 (33.2)	-	57 (17.6)	93 (28.7)	18 (5.6)	21 (6.5)	24 (7.4)	6 (1.9)	33 (10.2)
Mid back problem	42 (12.9)	93 (28.6)	-	147 (45.2)	39 (11.9)	54 (16.7)	23 (7.1)	8 (2.4)	62 (19.0)
Neck problem	68 (20.9)	129 (39.7)	115 (35.3)	-	67 (20.6)	57 (17.6)	19 (5.9)	0.0	19 (5.9)
Headache	9 (2.8)	72 (22.2)	72 (22.2)	144 (44.4)	-	36 (11.1)	0.0	0.0	36 (11.1)
Shoulder problem	19 (5.8)	69 (21.1)	0.0	188 (57.9)	51 (15.8)	-	17 (5.3)	18 (5.5)	69 (21.1)
Hip symptom or complaint	13 (4.0)	100 (30.8)	50 (15.4)	75 (23.1)	0.0	25 (7.7)	-	0.0	75 (23.1)
Health maintenance or general check-up	37 (11.4)	79 (24.3)	35 (10.8)	53 (16.2)	35 (10.8)	26 (8.1)	0.0	0.0	70 (21.6)
Other	29 (9.0)	100 (30.8)	58 (17.9)	93 (28.6)	37 (11.3)	44 (13.8)	19 (6.0)	5 (1.6)	41 (12.6)

Results reported as proportions (%) within the main reason for presentation

**Fig. 3:**
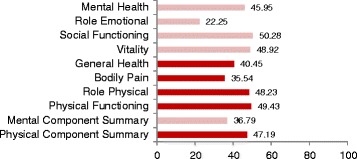
Summary of Short Form-12 mean scores. Societal Means: Physical Component Score (49.63) Mental Component Score (49.37)Pink bars represent domains included in the Mental Component Score, and red bars represent domains included in the Physical Component Score

†*The secondary complaints presented in the table are those most frequently recorded for the listed main reasons for presentation Conditions could be flagged multiple times by each patient*, *therefore total may* ≠ *100* %
  Table 6 and Fig. 3 show the scores for the SF-12 and PIQ6-R HQoL measures. The mean Physical Component Summary (PCS) was 47.19 (SD ± 8.46), and mean Mental Component Summary (MCS) was 36.64 (SD ± 7.92), compared with population mean scores of 49.63 and 49.37 respectively [31]. Pain Impact Questionnaire-R mean scores were 54.60 (95 % CI; 53.32–55.88), where the societal mean is 50.0 (SD ± 10) [38].

**Table 6:** Short Form−12 and Pain Impact Questionnaire scores (Stratified by age and location)

	Number of patients (*n*)	SF12 physical component summary	SF12 mental component summary	Pain impact score
		Mean (SD)	Mean (SD)	Mean (SD)
Age group				
18–34	174	46.79 (8.64)	36.72 (8.13)	54.14 (11.49)
35–54	121	47.57 (8.82)	36.84 (7.89)	54.57 (12.95)
55–74	30	48.04 (5.48)	36.94 (6.95)	57.37 (7.88)
Total	325	47.19 (8.46)	36.79 (7.92)	54.60 (11.79)
Practice location				
Inner Metro	223	47.59 (8.51)	36.45 (7.88)	53.07 (12.50)
Outer Metro	78	46.73 (6.58)	37.96 (7.21)	59.21 (7.58)
Regional	24	44.99 (12.51)	36.14 (10.18)	53.79 (12.78)
Total	325	47.19 (8.46)	36.79 (7.92)	54.60 (11.79)

Score values are shown as mean (± standard deviation)

## Discussion

This was the first study of its type in Western Australia that describes adult new patients who seek chiropractic care, and their quality of life indices. Though not a new approach, to our knowledge this study was the first in Australia to gather data from chiropractic patients electronically in this context. In some cases a pilot study will be the first phase of the substantive study and data from the pilot phase may contribute to the final analysis; this can be referred to as an *internal* pilot. Or at the end of the pilot study the data may be analysed and set aside as in the case of our study and thus is a so-called *external* pilot [28].

### Patient characteristics

Our data confirmed that the majority of patients (33.2 %) consulted chiropractors for low back pain (LBP) followed by neck pain (NP) (20.9 %) and mid-back pain (MBP) (12.9 %). Western Australians who consulted our chiropractors overwhelmingly spoke English as their first language, were employed and privately insured, paid all or part of the fee “out of their own pocket”, and presented with a non-specific spinal musculoskeletal problem. These findings are broadly consistent with previous studies [4, 12].

### Source of patients

Our results also confirm word of mouth and personal recommendation is the most important method of attracting patients. As there is evidence that the public and people with spinal pain know very little about either spinal pain or who treats it or where to access services [39, 40], we believe this means that public awareness and promotion will still be an important part of marketing and public relations for the profession in the future.

### Presenting complaints

Self-reporting by patients has been shown to be a reliable method of data collection in this context [32, 33] which is important given the emerging importance of patient-centered care and consumer driven health system planning [41]. While much of the focus in spinal pain research and subsequent guidelines to date has been on low back pain, [42] the current data highlight the importance of neck and mid-back problems since the proportion of patients presenting with neck and mid-back in combination (46 %) was actually greater than that of low back problems (33.2 %). These findings also confirm for example, a Danish study of 34,902 adults which concluded that back pain and its consequences may be regarded as the same condition regardless of where the pain happens to be located [43]. There is also an increasing recognition of syndromes such as “text neck” which describes the posture formed by forward head carriage over a mobile phone or other portable electronic devices while reading and texting. Prolonged use of these now ubiquitous technologies may increase the likelihood that such patients may consult chiropractors [44].

We found that the majority of people present with ‘multi-site’ problems. Even people who indicate they are presenting for health maintenance or a ‘general check-up’ usually report multiple problems. This is clinically relevant given the documented poorer prognosis of patients presenting with multiple sites of pain [45]. One of the most noteworthy features of these data is in the prevalence of co-existing (other area) spinal problems. Of the secondary problems reported, neck problems are the most common, particularly for those with low back or mid back, headaches and shoulder problems.

Whilst a majority of patients report no major co-morbid conditions, new patients who present to chiropractors may indeed have previously been diagnosed with potentially serious health conditions. Whilst there was some evidence of patient referral between GPs and chiropractors, this was apparent in only a very small proportion (1.8 %). The chiropractor is thus often acting in a ‘primary contact’ role for people with spinal problems. Just under half of patients reported previously consulting other chiropractors (48.6 %), thus practitioners need to identify both previous responses to management and also ensure that relevant clinical information is not overlooked. It is consequently vital that chiropractors follow evidence-based clinical decision-making protocols [46] and adhere to clinical practice guidelines for managing patients and also be alert to the more serious causes of spinal pain outside of their scope of practice [47, 48]. It is known that a higher level of multiple somatic symptoms is associated with poor health and work ability in patients with LBP [49]. The very first consideration in clinical decision-making is to ensure there is no delay in recommending more appropriate care [48]. These are important insights with implications for chiropractic education, practice, continuing professional development (CPD), and activities of representative organisations.

### Mental health issues

The proportion of persons presenting with a self-report of diagnosed depression is low, which contrasts with the low Mental Component Scores (MCS) scores. This is important for several reasons. There is an established association between back pain and low indices of social and emotional well-being, and the importance of managing these patient populations within a biopsychosocial model has been well documented [50–52]. The association with depression is supported by the low MCS scores reported our study where 86 % of respondents score below societal norms; along with 76 % being positive for first stage depression screening risk. These data are consistent with previous results, for example Coulter [16] found that compared with medical back pain patients, chiropractic back pain patients in North America had significantly worse mental health. These findings are extremely important in the context of studies linking depression, anxiety and chronic pain [53]. Questions concerning the role chiropractors already play as primary contact clinicians and in future multidisciplinary care within the context of spinal pain and the WA SPMoC [54] will need to be addressed by the profession.

The Council on Chiropractic Education Australasia (CCEA) lists a number of elements of competency for registration as a chiropractor including understanding of the significance of the major risk factors for disease such as obesity, poor nutrition, alcohol abuse, drug abuse, stress, mental health disorders, smoking, exposure to harmful environmental factors, and poor hygiene, the most common mental health disorders, and best practice treatment for these disorders. The Council also expects chiropractors to recognise the role that they can play in overall public health practice, including the public health system [55]. Thus chiropractors must be especially vigilant with respect to the social and emotional well-being of their patients and be mindful of current clinical practice guidelines which recommend the provision of advice and information addressing unhelpful beliefs, fear avoidance and to promote self-management [56].

### Consultation for preventive health issues

We found that 11.4 % of patients listed either ‘health maintenance’, ’prevention’ or ‘general check-up’ as a main initial reason for consultation. Further, these reasons for attendance were frequently listed as an *additional* reason for consultation. There is an ongoing debate in the chiropractic profession regarding ‘chiropractic wellness care’ [57] and the belief that regular chiropractic care may have value in maintaining and promoting health [58]. Although only a minority of people consult chiropractors initially for these reasons, they represent both a responsibility and opportunity for the profession to be equipped and proactive in an evidence-based health promotion role. Mainstream evidence-based health promotion strategies, such as smoking abstinence/cessation and weight reduction education, alongside spinal care, have already been endorsed by a 2011 consensus process within the chiropractic profession [57] although little data exist with respect to their uptake. If chiropractors feel they are ‘clinicians’ rather than ‘therapists’ then there needs to be commensurate depth and breadth to chiropractic education and practice, which includes expertise in the implementation of Evidence-Based Practice (EBP) and clinical competence in the implementation of evidence-based health promotion and expert spinal syndrome management that goes far beyond manual spinal care.

### Implications

#### Chiropractors as primary healthcare clinicians

These data suggest that the adults who consult chiropractors are in the main relatively healthy, who have non-malignant spinal pain, and who are working professionals or in managerial roles and thus likely to be well-informed about their healthcare needs. For the most part, chiropractors may not see complex patients with multiple co-morbidities and/or polypharmacy, and a relatively limited number of elderly patients (> 65 years) (we did not record those people < 18 years). By comparison, the average Australian General Practitioner (Family Physician) must have a good working knowledge of over 160 problems to cover 85 % of the conditions they will see most frequently, across a range of age groups, and be able to manage these disorders competently [59, 60]. Consequently, this kind of data regarding medical practice informs undergraduate medical curricula and post-graduate professional development, focusing on teaching/learning strategies covering the common diagnoses/problems. The implication is that if the role of chiropractic were to expand beyond that of caring for essentially healthy patients with non-malignant spinal pain and be competent to engage in the multi-disciplinary management of more complex patients, as encountered in the broader primary healthcare community, then educators, teachers and professional bodies need to ensure educational curricula equip chiropractors with these knowledge, skills and clinical attributes.

#### Insights gained from the synthesis of the results

Drawing upon outcomes of this study, themes emerge that correlate remarkably well with recommendations offered in Models of Care [54] and Pain Health Frameworks. Both these works being the output of the Musculoskeletal Network of the Western Australia Department of Health, respectively. These themes are; patient education and triage, multi-disciplinary, multi-modal care, and future education and training of healthcare professionals.

Chiropractors may well be able to integrate and offer care services as a team-member of mainstream care services, *if* they are prepared to develop inter-professionally and evolve their professional relationship skills along the lines of the themes presented.

The insights and recommendations generated by this pilot study will consequently be useful to inform fully powered, funded studies such as may occur for example under the auspices of the Australian Chiropractic Research Network (ACORN) project [11, 61].

### Strengths and Limitations

The strengths of this external pilot study are that it enabled some description of characteristics of (adult) people who consult chiropractors in WA. It also allowed estimation of adequate sample size for a larger study, with a low level of missing data. It had a high patient response rate, demonstrating ease of setting up data collection and with good breadth of data capture. The experiences and outcomes of this study thus support the feasibility of a future confirmatory study. Limitations include the lack of data collected from rural and remote locations despite several practices being recruited, therefore we were unable to analyse differences by geographical location as planned. We did not collect data from people under age 18, so cannot comment on this subset. In addition a large proportion of the data were collected in one large multi-practitioner inner city practice.

## Conclusions

This study provides insightful data regarding chiropractic patients and practices in Western Australia. The average chiropractic patient has musculoskeletal problems, with a high proportion presenting with pain (mainly spinal) at multiple sites. A significant number of patients also had co-existing mental health issues, particularly depression. Whilst chiropractors already have an important role to play in the evolving healthcare system in Western Australia, there may need to be professional and educational development to prepare and equip them for expansion into public sector roles.

## Abbreviations

(C) SMT, (Chiropractic) spinal manipulative therapy; AHPRA, Australian Health Practitioners Regulation Agency; CBA, Chiropractic Board of Australia; CCEA, Council on Chiropractic Education Australasia; HQoL, Human Quality of Life (measure); LBP, Low back Pain; MBP, Mid back Pain; MCS, Mental Component Score; NP, Neck Pain; PCS, Physical Component Score; PIQ, Pain Impact Questionnaire; PISTACHIO, Practice-Based Investigation and Study of Attendees at Chiropractic Clinics; SF, Short Form (12 & 36); WA SPMoC, Western Australian Spinal Pain Model of Care.

## Appendix 1

**Qualitative Responses** (with examples)

Positive Responses

Practitioners provided 90 % of the positive comments regarding the study, 45 % were positive attitudes toward research and several commended the team on the study. 27 % commented that the study had a low impact on the normal clinic operations.

“If this simple form of data collection can provide the Chiropractic profession with valuable data regarding the delivery of its services it is hard to understand why we have taken so long to begin it. Well done to the research team” (Chiropractor)

“Will always help with chiropractic research to the best of my ability” (Chiropractor)

“Thanks for including our clinic. Best of luck with the study” (Chiropractor)

“As a chiropractor I felt no impact on the running of the clinic and patients seemed very happy to be involved” (Chiropractor)

“Study seemed to go well, participants all keen to fill out” (Support staff)

Neutral Responses

The comments categorised as neutral expressed a benign perspective on the study and were the smallest category proportionally (7 %). Respondents typically felt the study had little impact on the clinic and reflected the lack of impact on practitioners who provided most of these responses. Also included in this section were neutral comments on ‘technology challenges’.

“The survey didn’t really affect me in any way as it was all dealt with at the front desk” (Chiropractor)

“Was unable to perform the study without iPad in office. Then went through a couple of weeks where I forgot about the study and therefore didn’t ask several patients to participate. Also had technical difficulty with the iPad” (Chiropractor)

“Didn’t really effect operations in the practice which was good, but maybe provide more information to the participants and us as they were asking us questions” (Support staff)

Negative Responses

The largest category of response was negative (*n* = 34) and came mainly from support staff (69.33 % of category). The 2 main issues that were highlighted were time (32.35 %) and length of survey (20.59 %) which could possibly be considered as different ways of expressing the same sentiment.

“Often caused participants to be approx. 5–10 min late for their appointment due to filling out lengthy form…. Most participants commented on the length of the form and would have preferred just one page. Option for printed forms could have increased compliance. No data is given to chiropractor therefore follow-up not possible. Would have been easier to comply with a receptionist” (Chiropractor)

Too time consuming, interfered with patient appointment times, created stress for the staff. Patients felt overwhelmed. (Support staff)

Took a lot of extra time not always easy when trying to keep clinic going at right pace (Support staff)

Patients didn’t like having to fill out multiple items.... Our intake form + the survey + our feedback form (Support staff)

“In our clinic I feel this survey caused a large inconvenience it sometimes meant new patients been seen late and patients feeling overwhelmed due to our required paperwork then adding this survey on top” (Support staff)

“Biggest hurdle was the time it was taking new patients to fill out existing paper work with the addition of the research questionnaire-which sometimes meant we ran late getting new patients into the consult rooms” (Support staff)

Suggestions

Suggestions for future improvements mostly related to the disclaimer and the length of the survey, although expressed in a less negative way than those in the ‘negative’ category.

“It was difficult giving it [survey] to people when we didn’t have any relationship with them at all. May have been easier to give it to them on the second or third visit” (Support staff)

“Don’t have such a long disclaimer. Took patients too long to read it and then begin the survey before their appointment” (Support staff)

“Survey time to be as minimal as possible” (Chiropractor)
